# Numerical Investigation on the Aerodynamic Benefits of Corrugated Wing in Dragonfly-like Hovering Flapping Wing

**DOI:** 10.3390/biomimetics10050256

**Published:** 2025-04-22

**Authors:** Arun Raj Shanmugam, Chang Hyun Sohn, Ki Sun Park

**Affiliations:** 1 Department of Mechanical and Aerospace Engineering, United Arab Emirates University, Abu Dhabi 15551, United Arab Emirates; arunraj.v2009@gmail.com; 2 School of Mechanical Engineering, Kyungpook National University, Daegu 41566, Republic of Korea

**Keywords:** aerodynamics, corrugation, dragonflies, flapping wing, hovering, insect flight

## Abstract

The effect of corrugated wings on the aerodynamic characteristics of a dragonfly-like hovering flapping wing is investigated using two-dimensional numerical simulations. Two types of pitch motion profiles, namely ‘sinusoidal’ and ‘trapezoidal’, are employed. The results obtained from the corrugated wings at Reynolds number Re = 2150 are then compared with the flat plate geometries to analyze the aerodynamic benefits of wing corrugation. The aerodynamic characteristics of corrugated wings are investigated quantitatively using cycle-averaged vertical force coefficient. For the qualitative investigation, time histories of vertical force coefficient, vorticity, and surface pressure distribution are used. The results reveal that the corrugated wings perform better than the flat plates in all three flapping configurations for both sinusoidal and trapezoidal pitch profiles. For a tandem wing with a sinusoidal pitch profile, the corrugated wings yield a vertical force generation nearly 14%, 22%, and 12%, higher than the flat plate geometries for ψ = 0°, 90°, and 180°, respectively. The corrugated wing sheds a relatively stronger detached counter clockwise vortex (CCWV) on the lower surface as compared to the flat plate, and hence, the vertical force is much higher for the corrugated wing. For a tandem wing with a trapezoidal pitch profile, the corrugated wings yield a vertical force generation nearly 27%, 22%, and 57%, higher than the flat plate geometries for ψ = 0°, 90°, and 180°, respectively. In corrugated wing geometry, the delayed stall mechanism is slightly postponed due to the corrugation shape’s ability to trap the vortex structures, leading to a positive effect on vertical force production.

## 1. Introduction

The flow over flapping wings, at the scale of insects, is an important topic due to its broad applications in micro aerial vehicles (MAVs). Numerous experimental and numerical studies have been performed on the fluid dynamics of natural flyers including insects [1,2,3,4,5,6] and swimmers [7,8,9,10,11,12,13,14]. Flapping wing flight, observed in flying insects, has been more aerodynamically efficient in comparison to fixed wing flight [15,16,17]. This has inspired researchers to mimic the wing kinematics and wing geometry of efficient flyers and swimmers [7,8,9,10,11,12,13,14,18,19].

Insect flight is one of the most important areas of research in science and engineering. The aerodynamics of insect flight is complex and it has applications in various areas, including defense. The flight strategies of insects can be classified into three types based on the force generation mechanisms: lift-based, drag-based, and hybrid. In the lift-based strategy, the wings flap along the horizontal plane to increase the lift force, as observed in insects such as fruit flies, bees, and wasps [20,21,22]. In the drag-based strategy, the wings flap along the vertical plane to exploit the drag force for locomotion, e.g., butterflies and fruit flies [23,24,25]. In a hybrid strategy, the wings flap in an inclined stroke plane to exploit both lift and drag to generate the vertical force, as observed in insects such as beetles, dragonflies, and hoverflies [26,27].

Dragonflies are prominent creatures, widely recognized for their outstanding hunting skills, astonishing agility, endurance, and maneuverability. They have expertise in flight ranging from sustainable hovering to backward flight and quick turns [28,29,30]. Dragonflies often fly at a low Reynolds number, flapping their wings at a high stroke plane inclination (β) of 60° and flapping frequency (f) of 20–40 Hz [31,32,33,34,35,36]. Dragonflies have four wings, two each mounted on either side of the thorax in tandem configuration, controlled and operated independently. Hence, there exists a phase difference between the forewing and hindwing, ψ, depending on the flight condition, to generate large aerodynamic forces [37,38,39].

In the past, multiple experimental and numerical studies were conducted to investigate the aerodynamic characteristics of dragonfly-like flapping wings in single and tandem arrangements using elliptical and flat plate geometries. Wang used numerical simulation to estimate that three-fourth of the vertical force generation in inclined stroke plane hovering comes from the drag [27,32]. Many unsteady aerodynamic force generation mechanisms including delayed stall, wake capture, and rotational circulation were found to play a crucial role in dragonflies’ flight [21,22,23,24,26,40,41,42]. Experimental and numerical simulations based on tandem flapping wings reveal that the forewing–hindwing vortex interaction has a huge role in the aerodynamic performance of dragonfly-like hovering flapping wings [43,44,45,46,47,48,49]. Tandem wing arrangement was found to perform better the single wing arrangement in vertical force generation, especially when flapping in-phase, implying aerodynamic benefits due to vortex interaction [34,44]. Besides dragonflies, the tandem wing was found to be aerodynamically beneficial in the fanning of honey bees [50]. Although numerous studies were carried out to understand the aerodynamic characteristics of dragonfly-like flapping wings, most of them used general wing shapes like elliptical, with limited studies on the corrugated wings observed in dragonflies.

Some studies evaluated the performance of corrugated wings in gliding flight [51,52,53]. Meng and Sun [51] reported that the wing corrugation decreases the lift generation in gliding flight. The wing corrugation caused low-pressure regions on the lower surface of the wing and strong flow separation on the upper surface that might lead to lift reduction. Hu and Tamai [52] showed experimentally that the aerodynamic performance of corrugated wings inspired by dragonflies was better than the streamlined shape and flat plate in gliding flight at low Reynolds number (Re). The wing corrugation can suppress the flow separation to enhance the performance. Shi et al. [53] elucidated the effect of camber on the performance of corrugated dragonfly wings in gliding flight. They found that an increase in maximum camber can improve the vertical force generation but it decreases the horizontal force generation.

Few other studies evaluated the performance of corrugated wings in normal hovering flight and propulsion mode [54,55]. Meng et al. [54] predicted that wing corrugation slightly decreases (~5%) the vertical force generation in normal hovering insects. The reason behind this decrement was caused by the strong flow separation at a large angle of incidence. Lian et al. [55] observed that corrugated wing shape does not provide an advantage in either stall delay or lift generation. However, corrugated wing shape was found to have structural advantages in comparison to flat plate. Flint et al. [56] investigated the effect of corrugated wing shape on the performance and flow characteristics around the pitching wing. They found that the corrugated wing did not show better propulsion efficiency than the smooth airfoils as the reduced frequency effect is a vital role for the wing performance. In recent times, some studies have been performed on corrugated wings for gliding and normal hovering flight [57,58]. Chitsaz et al. [59] found that corrugated wings can generate more lift-to-drag ratio as compared to flat plate by delaying flow separation and delayed stall.

Recently, Chitsaz et al. [60] revealed that the corrugated wing structures can act as a turbulator promoting boundary–layer transition, leading to delayed stall and improved aerodynamic performance. Sun et al. [3] studied the effects of unsteady motions of flapping flat plates and corrugated structures using numerical simulations. They found that the rigid corrugated wing could increase the vertical force generation by 10% in take-off acceleration flight when the advance ratio J is 0.36, as compared to flat plates. Hou et al. [61] investigated the aerodynamic performance of wing corrugation in inclined stroke plane hovering observed in dragonflies. The results showed that the wing with time-varying corrugations outperformed the rigid one in thrust generation and power consumption. The present literature indicates that few investigations have been conducted on the aerodynamic benefits of tandem corrugated wings, inspired by dragonflies, in inclined stroke plane hovering.

While three-dimensional simulations are important in capturing full aerodynamic behavior, such simulations are computationally expensive which makes them less suitable for this study. Therefore, two-dimensional simulations are often preferred over three-dimensional ones due to computational cost constraints. Many past studies on inclined stroke plane hovering have demonstrated that the two-dimensional models can effectively predict key flow features responsible for aerodynamic force generation with reasonable accuracy [20,27,32,33,34,35,36].

Against this backdrop, the present work aims to explore the aerodynamic benefits of wing corrugation in dragonfly-like hovering flapping wings at Re = 2150 using ANSYS Fluent V18. While a Reynolds number of 2150 is slightly high to be considered purely laminar, a previous study by Broering and Lian [62] showed the flow can be assumed to be a laminar model and no turbulence model is needed for Re < 5000. Two types of pitch motion profiles, sinusoidal and trapezoidal, are examined. Most numerical studies on dragonflies have used a sinusoidal pitch profile in the wing kinematics in which the pitch motion occurs during the entire cycle [7,20,27,32,33,34]. However, few others have used a trapezoidal pitch profile in the wing kinematics in which the pitch motion occurs only in the stroke reversal phase (supination and pronation) [63,64]. In this work, cycle-averaged force coefficients are used to characterize the benefits of wing corrugation quantitively. The qualitative analysis is performed using time histories of force coefficients, vorticity contour, and pressure distribution.

## 2. Numerical Methodology

**Figure 1:**
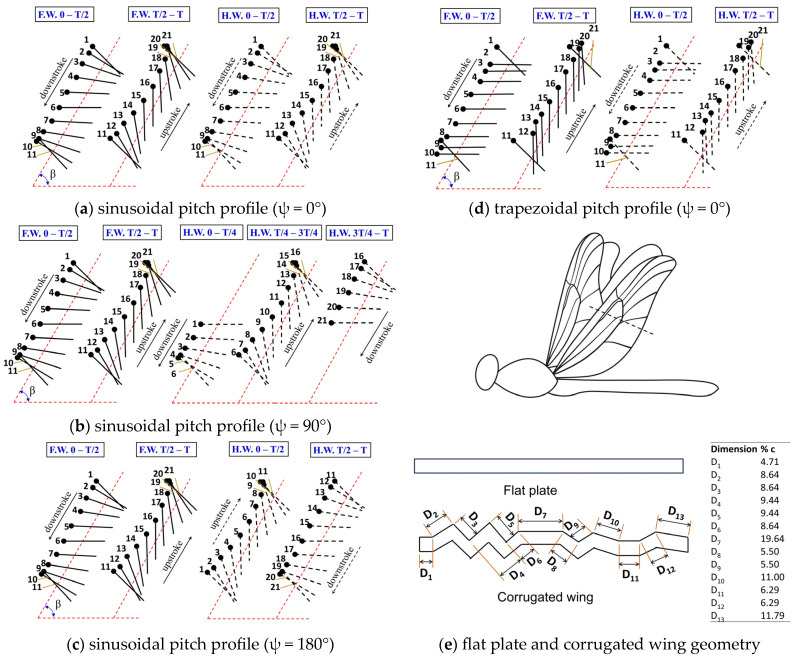
The wing kinematics of tandem flapping wing and wing geometry used in the study are shown. The FW indicates the forewing shown by solid line and HW indicates the hindwing shown by dashed line. T indicates non-dimensional time period. The sinusoidal pitch profile for three phase differences ψ = 0°, 90°, and 180° are shown in Figure 1(**a**–**c**). The trapezoidal pitch profile for a phase difference of ψ = 0° is shown in Figure 1(**d**). The corrugated wing geometry adopted from Kesel’s work [65] is shown in (**e**) along with their dimension in %chord.

The wing motion and wing geometry of the dragonfly-like flapping wing are shown in Figure 1. Various wing kinematic parameters used to model the wing motion are also marked in Figure 1. The flapping wing motion can be represented by two half time periods/cycles: downstroke and upstroke. Mathematically, the heave motion [x(t), y(t)] of forewing ‘FW’ and hindwing ‘HW’ is modeled using Equations (1)–(4). Two types of pitch motion profiles, sinusoidal and trapezoidal, are examined in this work. In the sinusoidal pitch profile, the pitch motion α(t) happens gradually for the entire duration of the cycle, as shown in Figure 1a–c. Meanwhile, in the trapezoidal pitch profile, the pitch motion α(t) happens mainly in the stroke reversal phase (supination and pronation), as shown in Figure 1d. The pitch motion α(t) of the forewing and hindwing is modeled with sinusoidal and trapezoidal profiles using Equations (5)–(8). A schematic of flat plate and corrugated wings studied here is reported in Figure 1e. The camber of the corrugated wing observed in dragonflies was not considered in the present work. Many previous studies have used elliptical wings [20,27,32,33,34,35,36]. However, the main goal of this work is to study how wing corrugation, seen in real dragonfly wings, affects aerodynamic performance during hovering. Since the corrugated wing is based on a flat plate shape, it makes more sense to compare it with a flat plate rather than an elliptical wing, which has a different geometry.

In real-life observations, three different phase differences are often observed in dragonflies, including in-phase stroking (ψ = 0°), 90° phase stroking (ψ = 90°), and counter stroking (ψ = 180°) patterns. For an in-phase stroking wing (ψ = 0°), the first half of a time period or cycle (t/T = 0–0.5) is a downstroke and the second half (t/T = 0.5–1) is an upstroke for both the forewing and the hindwing, as shown in Figure 1a. In the case of a 90° stroking wing (ψ = 90°), the first half of a time period (or cycle) is a downstroke and the second half is an upstroke for the forewing. However, for the hindwing, the first quarter (t/T = 0–0.25) and last quarter (t/T = 0.75–1) of a time period (or cycle) is a downstroke and the middle two quarters (t/T = 0.25–0.75) is an upstroke, as shown in Figure 1b. In the case of a counter stroking wing (ψ = 180°), the first half of a time period or cycle (t/T = 0–0.5) is a downstroke and the second half (t/T = 0.5–1) is an upstroke for the forewing. However, for the hindwing, the first half (t/T = 0–0.5) is an upstroke and the second half (t/T = 0.5–1) is a downstroke, as shown in Figure 1c.

Several assumptions are made in the numerical model, consistent with previous studies [20,27,32,33,34,35,36], for tandem flapping wings in inclined stroke plane hovering. The assumptions include the following:

1. The flapping wing trajectory is assumed to be linear, whereas real dragonflies exhibit a complex figure-eight motion, with the upstroke and downstroke motions following different trajectory.
2. The downstroke and upstroke durations are assumed equal, while in reality, the downstroke is significantly longer than the upstroke.
3. The wings are treated as rigid, without considering flexibility or twisting.
4. Three-dimensional effects are neglected.

The adopted wing kinematics is one of the simplest forms studied. The adopted wing kinematics model has some assumptions. However, it is found to be effective by many researchers to examine the aerodynamic characteristics of flapping wing in inclined stroke plane hovering observed in insects like dragonflies [20,27,32,33,34,35,36]. The initial position [x_i_, y_i_] and initial angle α_i_ of the wings can be computed from Equations (1)–(8) by substituting time t = 0. The pitch center x_p_ is located at c/3 from the leading edge (LE) of the hydrofoil. The wing motion parameters used in this work are shown in Table 1. The wing spacing L is measured horizontally between the two-stroke planes of the forewing and hindwing. The corrugated wing profile observed in dragonflies is adopted from the measurements made by Kesel (2000) to mimic the valleys [65].

Heave motion of forewing and hindwing is as follows:

(1) $$x_{f}\left(t\right)=\frac{A_{o}}{2}\;\cos\left(\beta_{f}\right)(1+\cos\left(\omega t\right))$$

(2) $$y_{f}\left(t\right)=\frac{A_{o}}{2}\;\sin\left(\beta_{f}\right)(1+\cos\left(\omega t\right))$$

(3) $$x_{h}\left(t\right)=\frac{A_{o}}{2}\;\cos\left(\beta_{h}\right)(1+\cos\left(\omega t+\psi \right))$$

(4) $$y_{h}\left(t\right)=\frac{A_{o}}{2}\sin\left(\beta_{h}\right)(1+\cos\left(\omega t+\psi \right))$$

where x(t) is the heave amplitude in the x-axis, y(t) is the heave amplitude in the y-axis, β is the stroke plane inclination, A_o_/c is the stroke amplitude in non-dimensional form, and ω = 2πf. Subscripts ‘f’ and ‘h’ indicate forewing and hindwing, respectively.

Pitch motion of forewing and hindwing for sinusoidal profile is as follows:

(5) $$\alpha_{f}\left(t\right)=\alpha_{o}–B\sin\left(\omega t+\varphi \right)$$

(6) $$\alpha_{h}\left(t\right)=\alpha_{o}–\;B\sin\left(\omega t+\varphi +\psi \right)$$

where α(t) is the pitch amplitude about z-axis, B is the pitch amplitude, φ is the heave-pitch phase difference, α_o_ is the mean angle of attack, ψ is the forewing–hindwing phase difference, and ω = 2πf.

Pitch motion of forewing and hindwing for trapezoidal profile is as follows:

(7) $$\alpha_{f}\;\left(t\right)=\alpha_{o}–\frac{B}{\tan h(C_{\alpha })}\;\tan h\;[C_{\alpha }\;\sin(\omega t+\varphi )]$$

(8) $$\alpha_{h}\left(t\right)=\alpha_{o}–\frac{B}{\tan h(C_{\alpha })}\;\tan h[C_{\alpha }\;\sin(\omega t+\varphi +\psi )]$$

where $C_{\alpha }$ is the pitch profile parameter ($C_{\alpha }\;$= 10).

The flow Reynolds number Re and pressure coefficient are given by Equations (9) and (10).

(9) Re=ρ ue cμ

(10) $$C_{P\left(\frac{x}{c}\right)}=\frac{P_{\left(\frac{x}{c}\right)}\;–{\;P}_{\text{∞}}}{0.5\;\rho u_{e}^{2}}$$

where u_e_ is the effective velocity (u_e_ = u_∞_ + u_f_) [66,67,68], u_∞_ is the free-stream velocity ($u_{\text{∞}}=0$ for hovering), u_f_ is the maximum flapping velocity (u_f_ = πfA_o_) [32,33,34], P_∞_ is the free-stream pressure, P_(x/c)_ is the surface pressure around the wing, x/c the x-position (chordwise), c is the chord length, ρ is the density of fluid, and μ is the dynamic viscosity of fluid.

The non-dimensional cycle-averaged vertical force coefficient $\bar{C_{v}}$ is given by Equation (11).

(11) $$\bar{C_{v}}=\frac{\int_{0}^{T}F_{v}\left(t\right)\;\mathrm{dt}}{0.5\;\rho {\;u}_{e\;}^{2}c\;T}$$

where $F_{v}\left(t\right)$ or $F_{v}$ is the instantaneous vertical force, $C_{v}\left(t\right)$ or $C_{v}$ is the instantaneous vertical force coefficient, and T is the time period. The symbol ¯ indicates the time-average.

**Figure 2:**
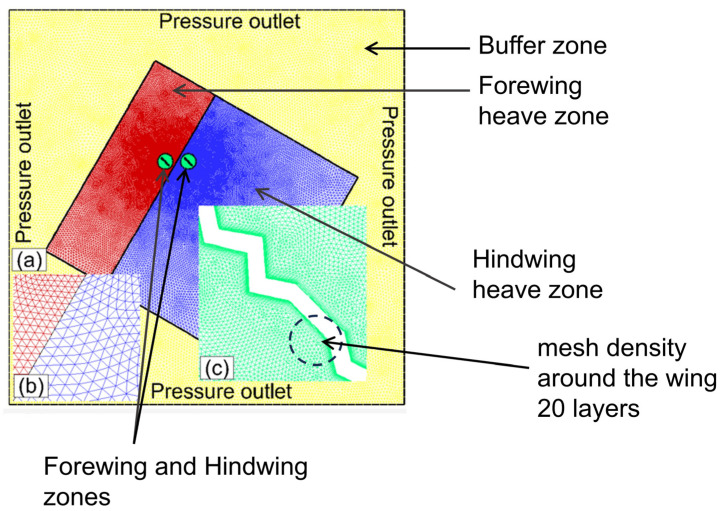
The computational domain (not drawn to scale), mesh model, and boundary conditions adopted for the simulation are shown. (**a**) computational domain (**b**) zoomed view of mesh near non-conformal sliding interface (**c**) zoomed view of mesh density around the wing. The forewing and hindwing zones are marked in green color. The forewing heave zone is marked in red color while the hindwing heave zone is marked in blue color. The buffer zone is marked in yellow color.

The computational domain and the boundary conditions applied for the numerical simulation are illustrated in Figure 2. The corrugated wing geometry used in the simulation has a thickness–chord ratio (t*/*c) of 4%. The flow Reynolds number Re is estimated as ~2150. The computational domain is a rectangle of length l (40c) and breadth b (36c). The pressure outlet P_∞_ is applied on all the boundaries in the hovering flight. A no-slip wall condition is imposed on the wing. Unstructured triangular meshes are created in this work and structured quadrilateral meshes are created around the wing to capture the boundary layer effects, as shown in Figure 2. The structured quadrilateral mesh grows at a rate of 1.1 to accommodate the required cells inside the boundary layer regime. The total number of mesh cells employed in the computational domain is typically around 1.50 × 10^5^.

An important challenge in the modeling of the flapping wing using the dynamic mesh method in ANSYS Fluent is the mesh deformation in the region surrounding the flapping wing. In this work, a specific approach was used to avoid the mesh deformation in the computational domain to further improve the accuracy of numerical results. For this purpose, the computational domain was subdivided into five zones: forewing zone, hindwing zone, forewing heave zone, hindwing heave zone, and buffer zone. A non-conformal sliding interface was used to ensure a smooth prediction of the flow. The forewing and hindwing zones executed ‘both heaving and pitching motion’ while the forewing heave and hindwing heave zones executed ‘pure heaving motion’. With this approach, the deformation in the mesh is restricted to mainly the buffer zone that is situated nearly 15c away from the wing. More details on this approach can be found in our previous study [69]. The spring-based smoothing and remeshing methods of the dynamic mesh technique were adopted to accomplish the mesh motion. During wing motion, the buffer zone undergoes smoothing and remeshing, according to the specified dynamic mesh settings. The spring-based smoothing method was used as it works better for the present study. The local remeshing option was applied in the buffer zone with a maximum skewness of 0.7 for the deformed mesh.

The flow field is solved using a pressure-based solver with a finite volume-based software ANSYS Fluent. The coupling between pressure and velocity is established with the Pressure Implicit with Split Operator (PISO) algorithm. The standard scheme is used to discretize the pressure term and the least square cell-based method is used to discretize the gradients. The spatial terms are discretized with a second-order upwind scheme. The unsteady terms are discretized with first-order implicit scheme. The flow around the foil is considered as unsteady, incompressible, and laminar. The governing equations of the fluid flow are shown in Equations (12)–(14).

Continuity and momentum equations are as follows:

(12) $$\frac{\partial u}{\partial x}+\frac{\partial v}{\partial y}=0$$

(13) $$\rho \left[\frac{\partial u}{\partial t}+u\frac{\partial u}{\partial x}+v\frac{\partial u}{\partial y}\right]=-\frac{\partial p}{\partial x}+\mu \left[\frac{\partial^{2}u}{\partial x^{2}}+\frac{\partial^{2}u}{\partial y^{2}}\right]$$

(14) $$\rho \left[\frac{\partial v}{\partial t}+u\;\frac{\partial v}{\partial x}+v\;\frac{\partial v}{\partial y}\right]=-\frac{\partial p}{\partial y}+\mu \;\left[\frac{\partial^{2}v}{\partial x^{2}}+\frac{\partial^{2}v}{\partial y^{2}}\right]$$

where u and v are the velocity components in x- and y-directions, respectively, and p is the pressure.

The mesh resolution is determined based on the results of the mesh independence test, as illustrated in Table 2. The effect of mesh resolution is studied for a hovering flapping wing with the following parameters: A_o_/c = 2.5, β = 60°, ψ = 0°, f = 40 Hz, and Re = 2150. The cases are summarized in Table 2. It can be seen from Table 2 that the change in $\bar{C_{v}}$ of the tandem wing becomes smaller between M2 (fine) and M3 (refined) and it is well within the acceptable limit (~2%). The mesh and time-step size of the fine case (M2) are deemed optimum, and hence, they are used for performing the subsequent simulations.

**Figure 3:**
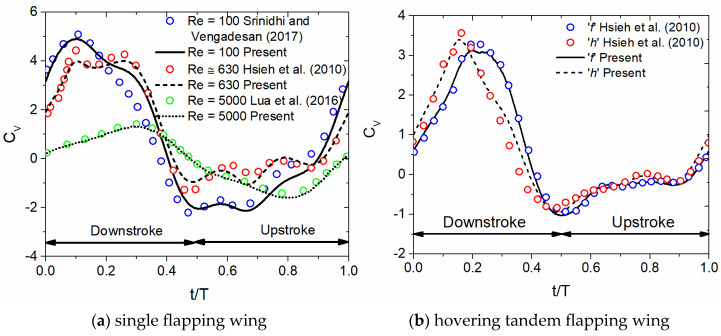
Comparison between the numerical results of the present study with the existing literature data. Srinidhi and Vengadesan [70] and Hsieh et al. [34] C_v_ data are obtained from numerical studies, while Lua et al. [71] C_v_ data are obtained from an experimental study. The letters ‘f’ and ‘h’ indicate forewing and hindwing.

Two validation studies are performed to ensure the credibility and validity of the numerical method employed in this work. The first validation study is the single flapping wing in an inclined or a vertical stroke plane for three different Re, as illustrated in Figure 3a. The first case has the following simulation conditions, A_o_/c = 2.5, β = 60°, f = 40 Hz, J = 0, and Re = 100, similar to the numerical data of Srinidhi and Vengadesan [70]. The second case has the following simulation conditions, A_o_/c = 2.5, β = 60°, f = 40 Hz, J = 0, and Re = 630, similar to the numerical data of Hsieh et al. [34]. The third case has the following simulation conditions, A_o_/c = 1.5, β = 90°, f = 0.67 Hz, and Strouhal number St = 0.32, similar to the experimental data of Lua et al. [71]. It can be observed from Figure 3a that in general, there is a good match between the present work and data (experimental/numerical) extracted from the literature at all three Re cases. However, there is a small difference noticed in C_v,_ especially in the middle of downstroke and upstroke, when compared with the data of Srinidhi and Vengadesan [70] at Re = 100.

The second validation study is the dragonfly-like hovering flapping wing in tandem arrangement, as illustrated in Figure 3b. The following simulation conditions, A_o_/c = 2.5, β = 60°, f = 40 Hz, ψ = 0°, L/c = 1.2, J = 0, and Re ≈ 630, similar to that of Hsieh et al. [34], are used. It can be noticed that the C_v_ of forewing ‘f’ is very close to the data of Hsieh et al. [34], while the C_v_ of hindwing ‘h’ indicates a slight difference near the middle of the downstroke [34]. In conclusion, the numerical model adopted in the present work is valid and reliable for studying dragonfly-like hovering flapping wings in tandem arrangement.

Due to the unavailability of experimental data of tandem flapping wings in inclined stroke plane hovering, the authors have validated the results of numerical model with the experimental data of Lua et al. [71]. Lua et al. [71] performed experiments on tandem flapping wings in vertical stroke plane for forward flight conditions. Our results agreed well with the data of Lua et al. [71], as shown in Figure 3a. There are many challenges in carrying out the experiments on tandem flapping wings in inclined stroke plane hovering in a controlled setup. It requires a recirculating water channel and sophisticated equipment such as digital particle image velocimetry DPIV systems, force sensors, and high-speed cameras, all of which involve significant costs. Accurately replicating the wing motions and the corrugated wing profile of dragonflies adds further difficulty.

## 3. Results and Discussion

The aerodynamic characteristics of a tandem flapping wing are dependent on the wing kinematics and wing geometry. This work examines the aerodynamic benefits of corrugated wings for the dragonfly-like hovering flapping wing. Three flapping wing patterns, in-phase stroking (ψ = 0°), counter stroking (ψ = 180°), and 90° phase stroking (ψ = 90°), are examined. Two types of pitch profiles, sinusoidal and trapezoidal, often used to model the dragonfly-like flapping wing motion, are investigated.

### 3.1. Hovering Tandem Wing with Sinusoidal Pitch Profile

In this section, the effect of a corrugated wing profile on the aerodynamic benefits of a dragonfly-like hovering flapping wing is explored using a sinusoidal pitch profile. In the sinusoidal pitch profile case, the corrugated wing continuously changes the pitch angle for the entire cycle, as shown in Figure 1a–c. The other wing kinematics parameters used for performing the simulations are as follows: A_o_/c = 2.5, α_o_ = 45°, β = 60°, f = 40 Hz, L/c = 2.1, J = 0, and Re = 2150. The wing spacing L/c used in this study was chosen based on the results of our previous study [44]. In general, in each case of the present work, a minimum of 10 flapping cycles were simulated to ensure that the force coefficients are periodically stable.

#### 3.1.1. Comparison of Vertical Force for Various Flapping Patterns with Sinusoidal Pitch Profile

It is well known that many insects including dragonflies generate large vertical force to counterbalance the weight during hovering. Dragonflies adjust their stroke plane and body inclination dynamically to nullify the horizontal force generation during hovering [70,72]. Therefore, studies on dragonfly-like hovering flapping wings have often neglected horizontal force in their studies [33,34,44,70,71]. But the flapping wing kinematics modeled using sinusoidal functions often lead to a net horizontal force due to asymmetry in the horizontal force generation of upstroke and downstroke. Following earlier works, the horizontal force generation was neglected in the present work as it is not vital for hovering flights.

**Figure 4:**
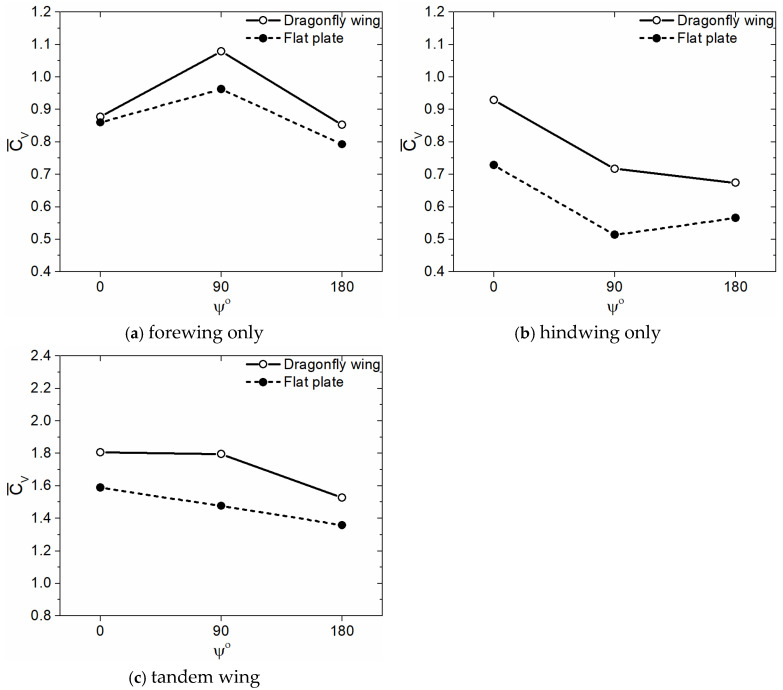
Comparison of mean vertical force coefficient ($\bar{C_{v}}$) of corrugated wing and flat plate for three different flapping patterns (ψ = 0°, 90°, and 180°) using sinusoidal pitch profile at Re = 2150. The forewing, hindwing, and tandem data are presented.

In this section, the aerodynamic benefits of corrugated wings for a dragonfly-like flapping wing with a sinusoidal pitch profile are investigated quantitatively using cycle-averaged values. The average vertical force coefficient $\bar{C_{v}}$ obtained for corrugated wings observed in dragonflies for three flapping patterns at Re = 2150, as shown by solid lines in Figure 4. The $\bar{C_{v}}$ of a flat plate is also shown by dash lines in Figure 4 as a baseline case for comparison purposes to evaluate the aerodynamic benefits of wing corrugation. Three different flapping wing patterns, in-phase stroking (ψ = 0°), 90° phase stroking (ψ = 90°), and counter stroking (ψ = 180°), are investigated. It is evident from Figure 4 that corrugated wing geometries outperformed flat plate geometries for the three investigated flapping patterns (ψ = 0°, 90°, and 180°). The corrugated wings have a positive impact on vertical force generation, which is essential for hovering flight.

Additionally, it is clear from Figure 4 that the vertical force generation depends strongly on the forewing–hindwing phase difference ψ. However, the mean vertical force coefficient $\bar{C_{v}}$ varies non-monotonically with phase difference ψ for both corrugated wing and flat plate geometries, as shown in Figure 4. As ψ varies between 0° and 90° for corrugated wing geometries, the $\bar{C_{v}}$ of the forewing in Figure 4a increases significantly but the $\bar{C_{v}}$ of hindwing in Figure 4b decreases drastically. Hence, for corrugated wing geometries, the $\bar{C_{v}}$ of 90° phase stroking tandem wing (ψ = 90°) becomes slightly less than the $\bar{C_{v}}$ of the in-phase stroking wing (ψ = 0°), as shown in Figure 4c. Meanwhile, when ψ is set as 180°, the $\bar{C_{v}}$ of counter stroking tandem wing (ψ = 180°) in Figure 4c drops significantly as compared to the $\bar{C_{v}}$ of in-phase stroking and 90° phase stroking tandem wings. In conclusion, the vertical force generation of tandem wings is highest for an in-phase stroking pattern whereas it is lowest for a counter stroking pattern, as illustrated in Figure 4c. A similar trend is also observed for flat plate geometries in Figure 4c, except in the case of 90° phase stroking, wherein the drop in $\bar{C_{v}}$ (tandem wing) is even more high, causing $\bar{C_{v}}$ to drop linearly from 0° to 180°.

Irrespective of the flapping pattern with sinusoidal pitch profile, the $\bar{C_{v}}$ of tandem wing obtained for the corrugated wing geometries is always higher than the flat plate geometries, as shown in Figure 4c. The $\bar{C_{v}}$ of tandem wing obtained for corrugated wing geometries is nearly 14% (for ψ = 0°), 22% (for ψ = 90°), and 12% (for ψ = 180°) higher than the flat plate geometries. This indicates that the corrugated wing geometries are superior to the flat plate geometries. The reason behind the huge increase in vertical force generation is explained in Section 3.1.4 using vorticity field and surface pressure distribution.

#### 3.1.2. Time History of Vertical Force Coefficient C_v_ for Various Flapping Patterns with Sinusoidal Pitch Profile

**Figure 5:**
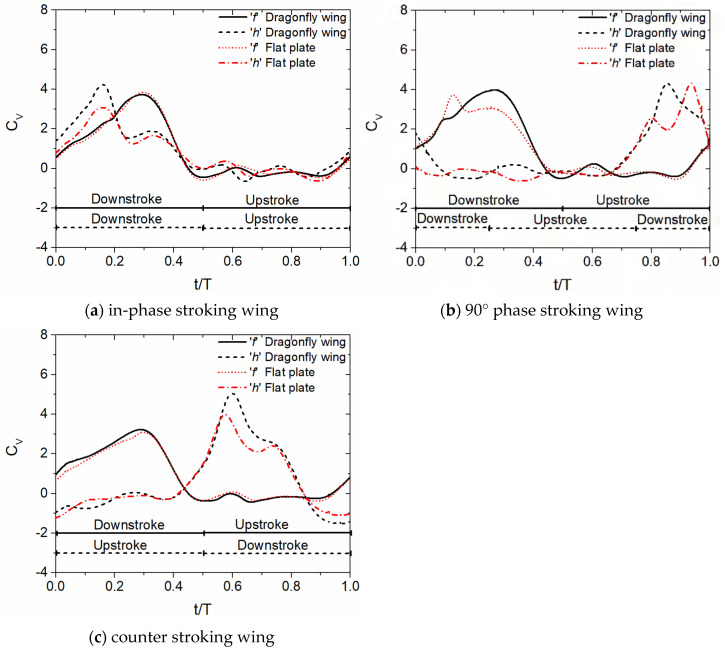
Time history of C_v_ for corrugated and flat plate tandem wings during inclined stroke plane hovering with sinusoidal pitching, shown for different flapping patterns (ψ = 0°, 90°, and 180°). The letters ‘f’ and ‘h’ denote the forewing and hindwing, respectively. The downstroke and upstroke intervals of forewing and hindwing for each flapping pattern are indicated at the bottom of the plot by solid and dashed lines, respectively.

The time history of the vertical force coefficient C_v_ of forewing and hindwing for three flapping patterns is illustrated in Figure 5. The non-dimensional time t/T is used to represent the flapping cycle. The upstroke and downstroke motions for each flapping pattern are also marked in Figure 5. The forewing’s upstroke and downstroke motions are marked using a solid line (at the bottom). Meanwhile, the hindwing’s upstroke and downstroke motions are marked using a dashed line (at the bottom). The letters ‘f’ and ‘h’ in Figure 5 represent forewing and hindwing, respectively. It can be seen from Figure 5 that most of the vertical force is generated in the downstroke. In addition, the forewing–hindwing vortex interaction has a major role in the vertical force generation of the forewing and hindwing. This vortex interaction is also strongly dependent on the forewing–hindwing phase difference ψ.

Firstly, let us compare the C_v_ of corrugated wing and flat plate geometries for the in-phase stroking (ψ = 0°) and counter stroking (ψ = 180°) patterns in Figure 5a,c. There is no notable increase observed in C_v_ due to corrugated wing geometry in the forewing for both in-phase and counter stroking, indicated by black solid lines in Figure 5a,c, as compared to the flat plate geometry, indicated by red dotted lines in Figure 5a,c. In the case of the hindwing, the corrugated wings outperform the flat plates in vertical force generation for both in-phase and counter stroking, especially in the downstroke intervals, as indicated by black dash lines and red dash-dotted lines in Figure 5a,c. However, the corrugated wing has a detrimental impact on the hindwing’s C_v_ in the interval t/T = 0.9–1 for the counter stroking pattern as compared to the flat plate, as shown by black dash lines and red dash-dotted lines in Figure 5c.

In the case of 90° phase stroking (ψ = 90°), a slight shift is noticed in peak C_v_ caused by the vortex evolution in corrugated wings and flat plates, as indicated in Figure 5b. Considering the forewing, the corrugated wings generate significantly more vertical force as compared to the flat plates, especially in the downstroke intervals, as indicated by black solid lines and red dot lines in Figure 5b. Meanwhile, in the hindwing, the corrugated wings generate slightly more vertical force as compared to the flat plates for most downstroke intervals, as indicated by black dash lines and red dash-dotted lines in Figure 5b. In conclusion, the corrugated wings are observed to perform better than flat plates in all three considered flapping patterns.

#### 3.1.3. Comparison of Flow Field for Various Flapping Patterns with Sinusoidal Pitch Profile

**Figure 6:**
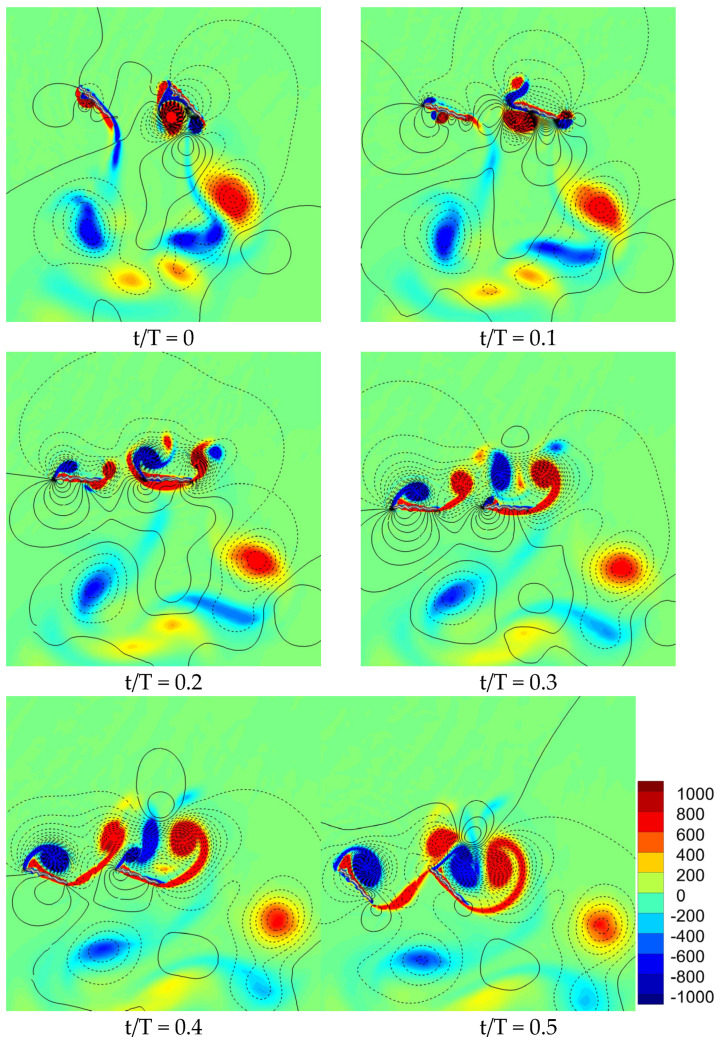
Snapshots of flow field (vorticity and pressure isoline) for an in-phase stroking wing. The positive pressure is shown by solid isoline while negative pressure is shown by dashed isoline.

It is well known that the vertical force generated by the flapping wing is strongly dependent on the evolution of vortex structures. Various mechanisms including the formation of clockwise vortex CWV and counter clockwise vortex CCWV, wake capture, and wing–wing interaction effect play a vital role in the vertical force generation. The flow field of a corrugated wing for in-phase stroking is illustrated in Figure 6. The time instant at which the flow field is predicted is also given in Figure 6. The vortex structures are shown by vorticity contour. The solid and dashed isolines in Figure 6 indicate positive and negative pressures, respectively. The clockwise vortex CWV and counter clockwise vortex CCWV are shown in blue and red color, respectively.

For every cycle of the in-phase stroking wing, two new vortices (CWV and CCWV) are formed on the upper surface of both the forewing and hindwing at the start of the downstroke (t/T = 0.1), as shown in Figure 6. However, the newly formed CWV and CCWV grow stronger for the hindwing as compared to the forewing, as shown in Figure 6. Meanwhile, on the bottom surface (t/T = 0–0.1), a detached CCWV exists for both wings, shed from the previous cycle. A stronger detached CCWV is present for the hindwing as compared to the forewing due to wing–wing vortex interaction and wake capture, as shown in Figure 6. Due to these reasons, the vertical force generated by the hindwing during downstroke is higher than the forewing, as shown in Figure 6.

**Figure 7:**
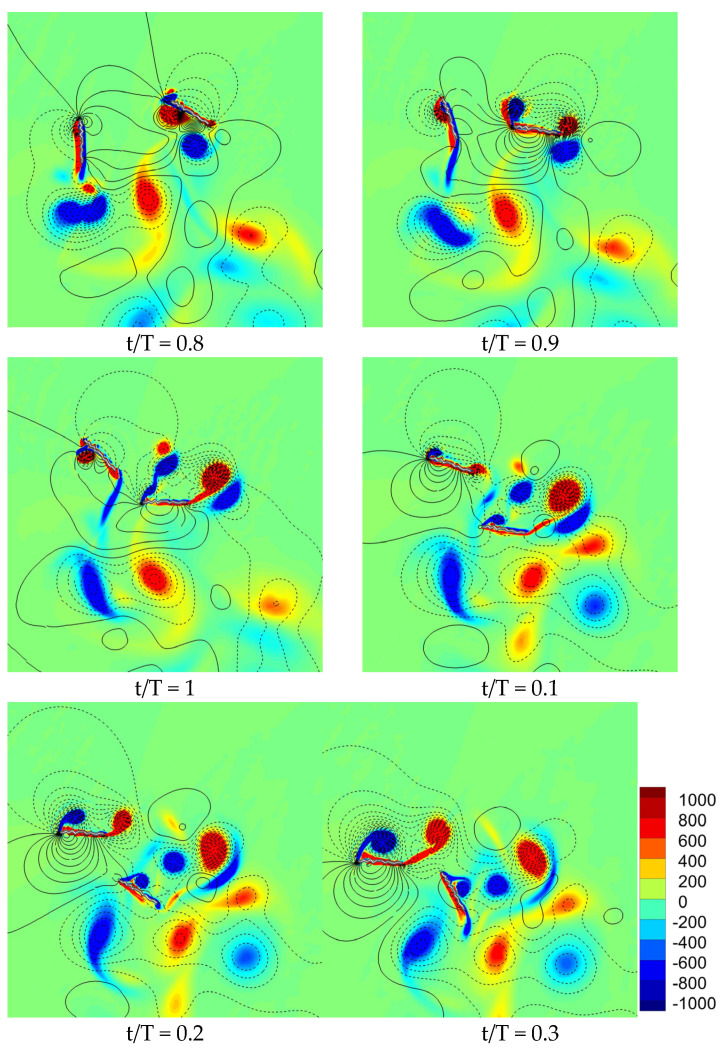
Snapshots of flow field (vorticity and pressure isoline) for a 90° phase stroking wing. The positive pressure is shown by solid isoline while negative pressure is shown by dashed isoline.

The flow field of a corrugated wing for 90° phase stroking is illustrated in Figure 7. The vortex structures of 90° phase stroking show a relatively weaker detached CCWV on the lower surface of the hindwing at t/T = 0.8, as compared to in-phase stroking. This detached vortex is relatively weaker with the introduction of a phase shift in motion due to its effect on the mechanism of wing–wing interaction. New CWV and CCWV vortices are formed on the upper surface of the hindwing in the interval t/T = 0.8–0.9. But the delayed stall occurs early for the 90° phase stroking as compared to the in-phase stroking. Meanwhile, for the forewing, much stronger CWV and CCWV vortices are newly formed on the upper surface (in the interval t/T = 0.1–0.3) for the 90° phase stroking as compared to the in-phase stroking. The formations of these new vortices are not affected by wing–wing interaction due to the introduction of phase shifts in motion. These new vortices will persist for a longer duration (in the downstroke) for the 90° phase stroking as compared to the in-phase stroking. Hence, the vertical force generation of the forewing is much higher for 90° phase stroking as compared to in-phase stroking.

**Figure 8:**
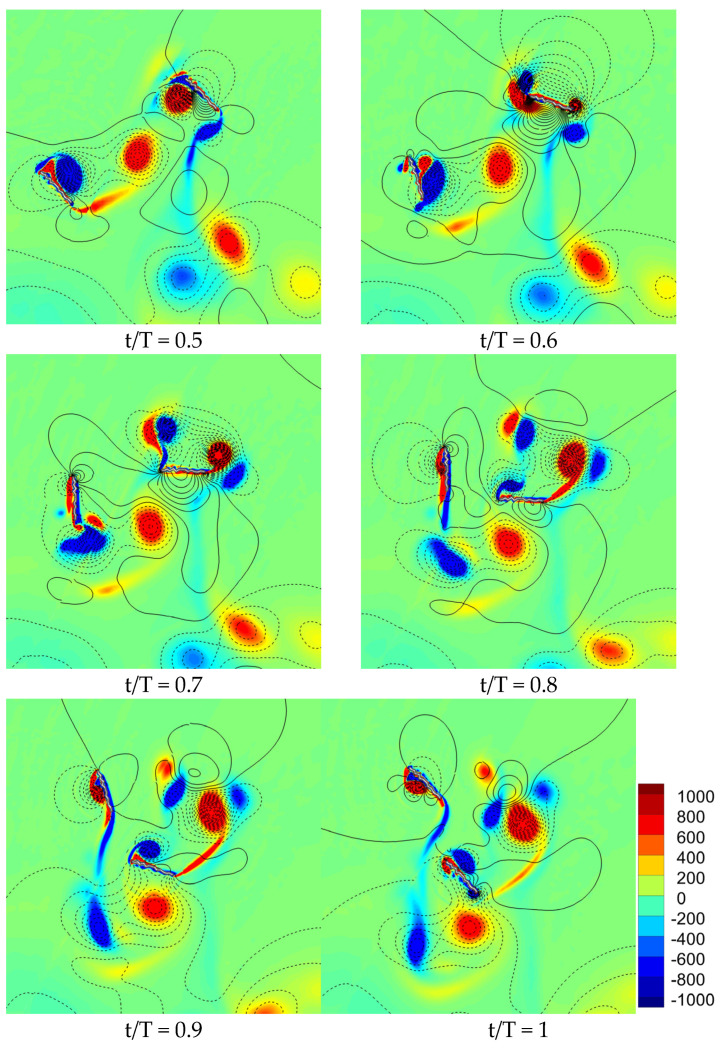
Snapshots of flow field (vorticity and pressure isoline) for a counter stroking wing. The positive pressure is shown by solid isoline while negative pressure is shown by dashed isoline.

The flow field of a corrugated wing for counter stroking is illustrated in Figure 8. The vortex structures of counter stroking show a relatively stronger detached CCWV on the lower surface of the hindwing during downstroke due to wake capture (in the interval t/T = 0.5–0.6), as compared to in-phase stroking. This vortex interaction will significantly enhance the vertical force generated by the hindwing during its downstroke. However, the hindwing also generates negative vertical force at the end of the downstroke when it interacts with the vortex from the forewing on the lower surface. From Figure 6, Figure 7 and Figure 8, it can be concluded that the wing–wing interaction effect has a destructive role on the hindwing’s vertical force generation for the counter stroking wing. Meanwhile, the wing–wing interaction effect has a constructive role on the hindwing’s vertical force generation for the in-phase stroking wing.

#### 3.1.4. Comparison of Flow Field for Corrugated Wings and Flat Plates Flapping with Sinusoidal Pitch Profile

**Figure 9:**
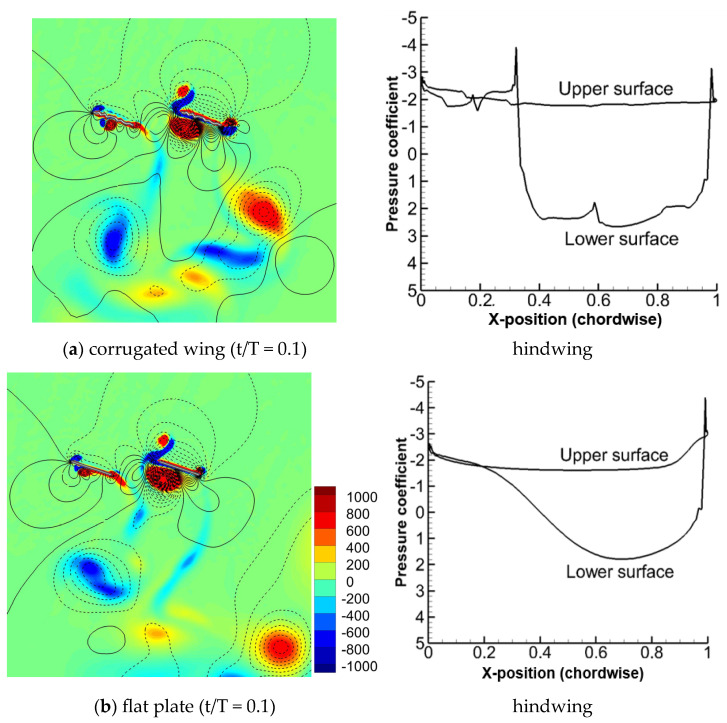
Comparison of vorticity and pressure distribution on the hindwing for corrugated and flat plate wings during in-phase stroking in a tandem wing configuration. The positive pressure is shown by solid isoline while negative pressure is shown by dashed isoline. The detached CCWV on the lower surface of hindwing is strengthened for corrugated wing as compared to flat plate. This is also evident from the pressure distribution plot shown on the right.

The flow field of in-phase stroking for the corrugated wing and a flat plate at t/T = 0.1 are compared in Figure 9. The pressure distribution of the hindwing for dragonfly wing and flat plate is also illustrated. Even though the core region of detached CCWV is found to generate negative pressure, its outer periphery generates a large positive pressure as it collides with the hindwing, as shown in Figure 9. The detached CCWV on the hindwing’s lower surface is relatively stronger for the corrugated wing as compared to the flat plate, as shown in Figure 9, and hence, its vertical force is high for the corrugated wing, as shown in Figure 5a. This detached CCWV is formed as a result of unsteady mechanisms by the fusion of vortices from wake capture and wing–wing interaction effects, resulting in a high-pressure region on the lower surface. This observation for a corrugated wing in inclined stroke plane hovering differs from the finding of Meng and Sun [51] at low Re, similar to this study, wherein the corrugated wing in gliding flight (stationary) causes a low-pressure region on the lower surface, possibly due to the absence of unsteady mechanisms, leading to lift reduction. Meanwhile, on the upper surface, the newly formed CWV and CCWV causes slightly more suction pressure for the corrugated wing as compared to the flat plate, which could be due to the trapping of vortices by the wing corrugation. In contrast, Meng and Sun [51] revealed that the corrugated wing in gliding flight pushes the leading-edge-separation layer, causing more flow separation, which then reduces the suction pressure and lift generation. However, the results from this study align with the study of Hu and Tamai [52], which was performed using a corrugated wing in gliding flight at a Re much higher compared to this study. Hu and Tamai [52] showed that corrugated wing discourages flow separation and stall, which was also observed in this study.

**Figure 10:**
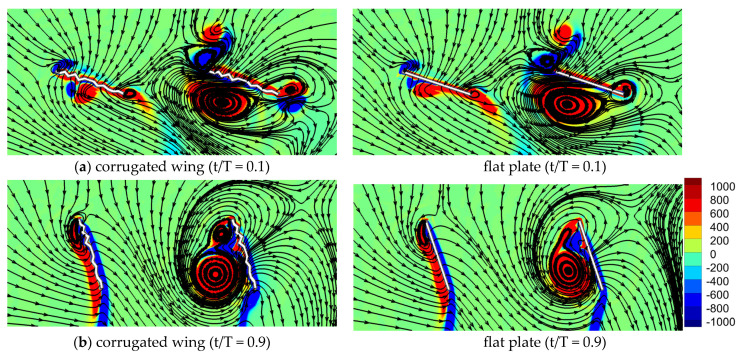
Comparison of vorticity and streamlines for corrugated and flat plate wings during in-phase stroking in a tandem wing configuration. At t/T = 0.1, a stronger detached CCWV is seen on the lower surface of hindwing for corrugated wing as compared to flat plate. At t/T = 0.9, the detached CCWV on the lower surface gains strength for corrugated wing as compared to flat plate.

The streamlines and vorticity of in-phase stroking for the corrugated wing and a flat plate are compared in Figure 10. The streamlines indicate that the flow features are quite similar for the corrugated wing and flat plate. As indicated in Figure 10a, a strong detached CCWV is observed on the lower surface, shed from the previous cycle. The streamlines on the suction side (upper surface) shown in Figure 10a are inward towards the wing as it is in downstroke. Meanwhile, the streamlines on the pressure side (upper surface) are shown in Figure 10b as outward to the wing as it is in the upstroke. Comparing the streamlines around the CCWV in Figure 10b, it can be noticed that the corrugated wing sheds a relatively stronger CCWV as compared to the flat plate.

**Figure 11:**
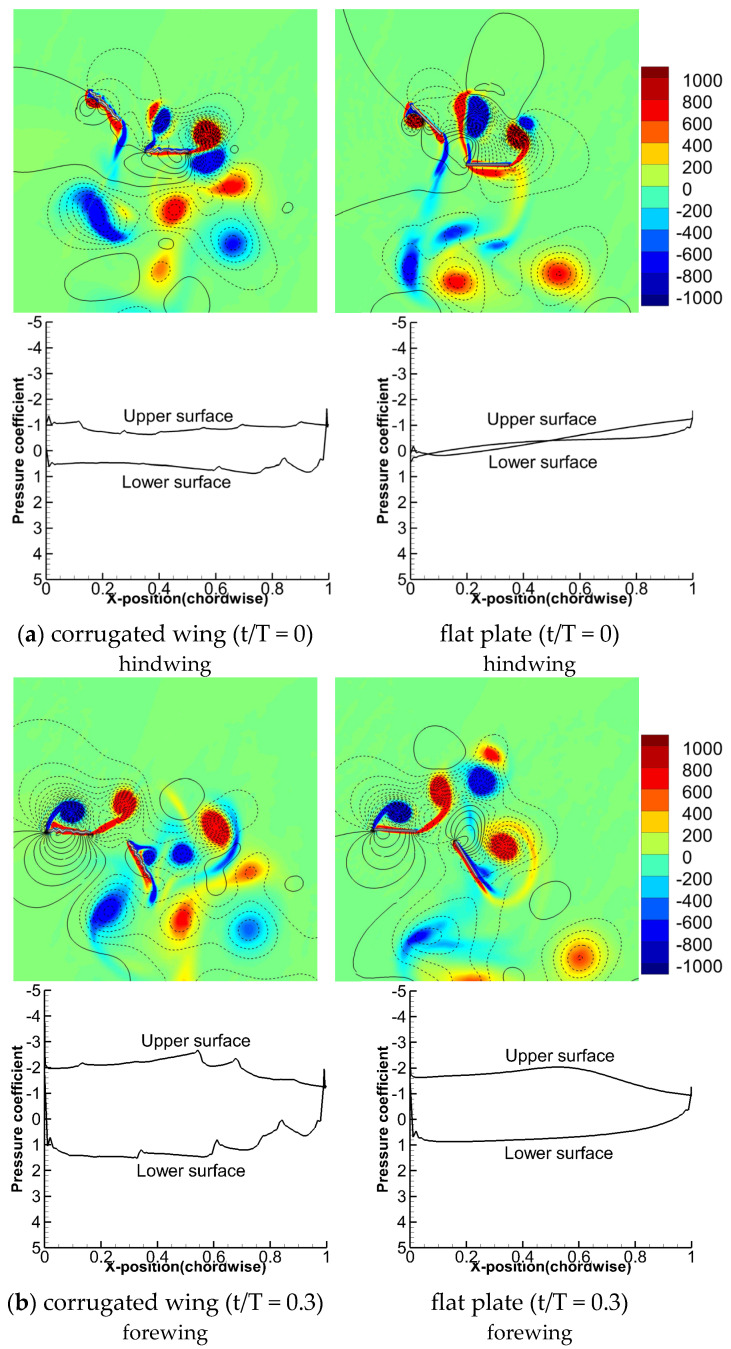
Comparison of flow field (vorticity and pressure isoline) and pressure distribution for corrugated wing and flat plate at ψ = 90°. The positive pressure is shown by solid isoline while negative pressure is shown by dashed isoline.

The flow field of 90° phase stroking for the corrugated wing and a flat plate at t/T = 0 and 0.3 are compared in Figure 11. The pressure distribution of hindwing at t/T = 0 and the pressure distribution of forewing at t/T = 0.3 are also shown. At t/T = 0, the hindwing is in the middle of the downstroke. Meanwhile, at t/T = 0.3, the forewing just crosses the middle of the downstroke. As indicated in Figure 5b, the hindwing’s vertical force is higher for the corrugated wing than the flat plate (at t/T = 0). The wing corrugation slightly delays the vortices shedding on the upper surface, causing a large surface pressure difference, as shown in Figure 11a. Meanwhile, the forewing’s vertical force is higher for the corrugated wing as compared to the flat plate (at t/T = 0.3). Relatively stronger vortices are formed on the upper surface of the corrugated wing that generates large negative pressure, as shown in Figure 11b. Meanwhile, the corrugated wing traps the detached vortices on the lower surface, generating large positive pressure as compared to the flat plate.

**Figure 12:**
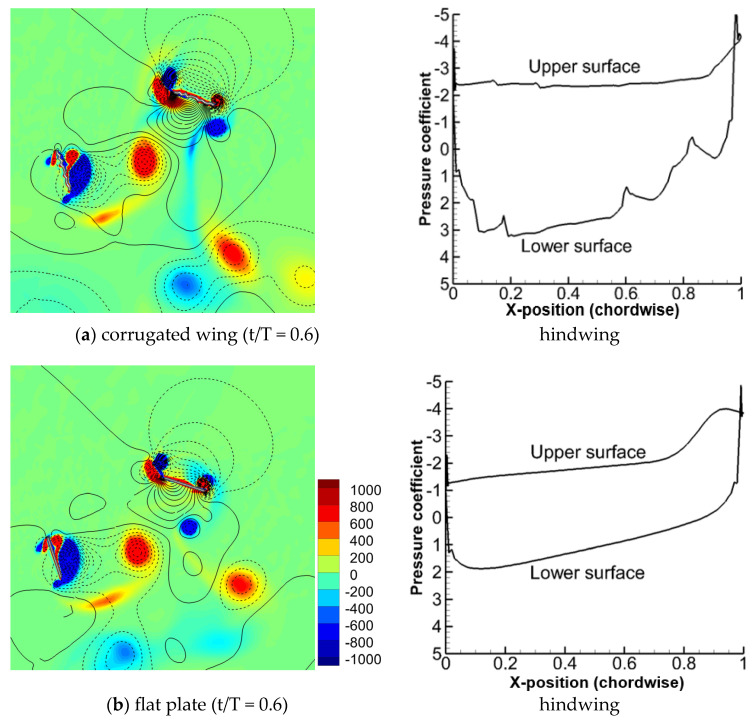
Comparison of flow field (vorticity and pressure isoline) and pressure distribution for corrugated wing and flat plate at ψ = 180°. The positive pressure is shown by solid isoline while negative pressure is shown by dashed isoline. A stronger detached CCWV is present on the lower surface of the hindwing for the corrugated wing as it traps the flow.

The flow fields of counter stroking for the corrugated wing and a flat plate at t/T = 0.6 are compared in Figure 12. The pressure distribution of the hindwing for the corrugated wing and flat plate is also shown. The upper and lower surfaces of the hindwing are the suction and pressure sides, respectively. As indicated in Figure 5c, the hindwing’s vertical force (in the downstroke) is higher for the corrugated wing than the flat plate. A stronger detached CCWV is present on the lower surface of the hindwing for the corrugated wing as it traps the flow. Meanwhile, a stronger CWV and CCWV on the upper surface of the hindwing cause relatively large negative pressure than a flat plate. These effects increase the surface pressure difference between the upper and lower surface for the corrugated wing as compared to the flat plate.

**Figure 13:**
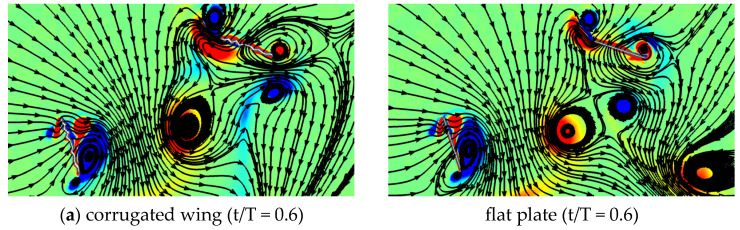

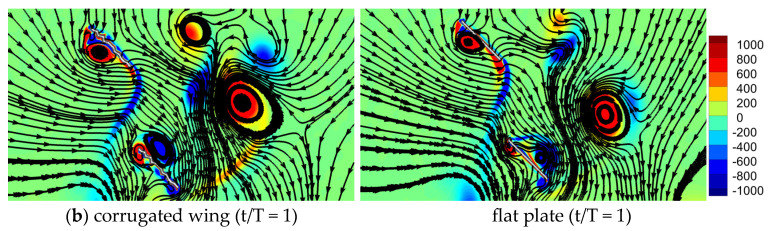
Comparison of vorticity and streamlines for corrugated wing and flat plate at ψ = 180°.

The streamlines and vorticity of counter stroking for the corrugated wing and flat plate are compared in Figure 13. A relatively stronger detached CCWV is observed on the lower surface at the start of the hindwing’s downstroke (t/T = 0.6), as indicated in Figure 13a. It is evident from Figure 13b that corrugated wing generates higher negative vertical force than a flat plate at the end of downstroke (t/T = 1) due to more destructive wing–wing interaction.

### 3.2. Hovering Tandem Wing with Trapezoidal Pitch Profile

In this section, the effect of a corrugated wing profile on the aerodynamic benefits of a dragonfly-like hovering flapping wing is explored using a trapezoidal pitch profile. In the trapezoidal pitch profile, the pitch motion happens only in the stroke reversal phase (supination and pronation), as shown in Figure 1d. The other wing kinematics parameters used for performing the simulations are A_o_/c = 2.5, α_o_ = 45°, β = 60°, f = 40 Hz, L/c = 2.1, J = 0, and Re = 2150.

#### 3.2.1. Comparison of Vertical Force Generation for Various Flapping Patterns with Trapezoidal Pitch Profile

**Figure 14:**
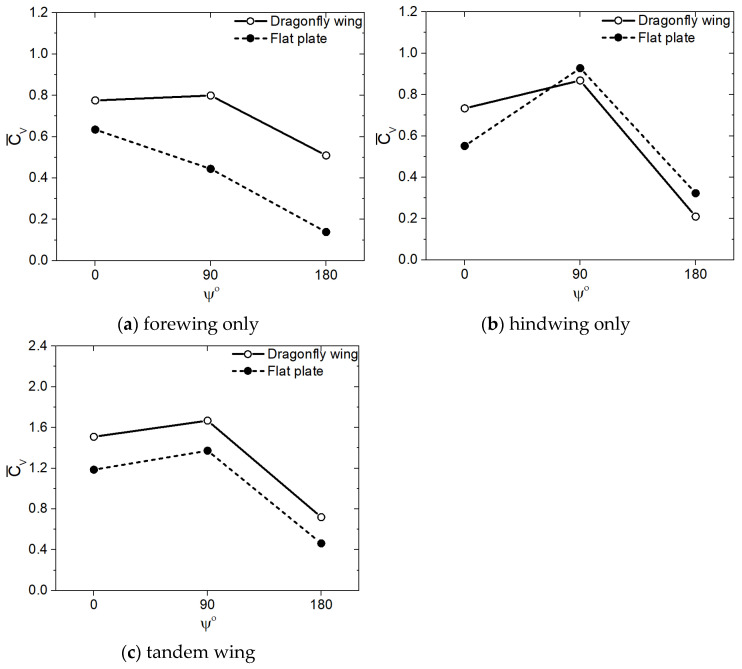
Comparison of mean vertical force coefficient ($\bar{C_{v}}$) of corrugated wing and flat plate for three different flapping patterns (ψ = 0°, 90°, and 180°) using trapezoidal pitch profile at Re = 2150. The forewing, hindwing, and tandem data are presented.

The average vertical force coefficient $\bar{C_{v}}$ obtained for the corrugated wings at Re = 2150 for trapezoidal pitch profile, as shown by solid lines in Figure 14. The $\bar{C_{v}}$ of a flat plate is also shown by dash lines in Figure 14. Three different flapping wing patterns, in-phase stroking (ψ = 0°), 90° phase stroking (ψ = 90°), and counter stroking (ψ = 180°), are presented. In all three flapping patterns with trapezoidal pitch profile, the corrugated wing geometries generate more $\bar{C_{v}}$ than the flat plate geometries, as shown in Figure 14c. The $\bar{C_{v}}$ of the tandem wing with trapezoidal pitch profile obtained for corrugated wing geometries is nearly 27% (for ψ = 0°), 22% (for ψ = 90°), and 57% (for ψ = 180°) higher than the flat plate geometries. Comparing the data of the sinusoidal pitch profile in Figure 4 and the trapezoidal pitch profile in Figure 14, it can be observed that a flapping wing with a sinusoidal pitch profile generates more vertical force than a flapping wing with a trapezoidal pitch profile. From Figure 4 and Figure 14, it can be concluded that the corrugated wing geometries are superior to the flat plate geometries for both sinusoidal and trapezoidal pitch profiles.

#### 3.2.2. Time History of Vertical Force Coefficient C_v_ for Various Flapping Patterns with Trapezoidal Pitch Profile

**Figure 15:**
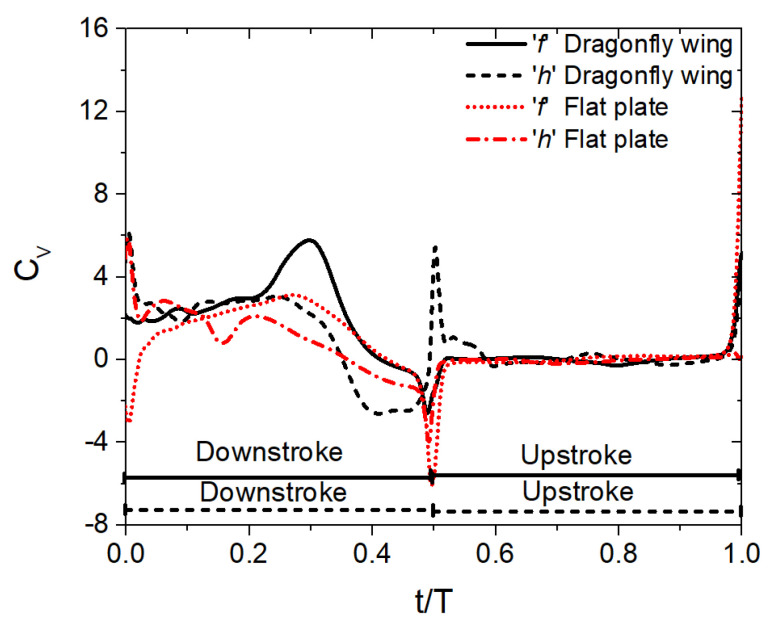
Time history of C_v_ for corrugated and flat plate tandem wings during inclined stroke plane hovering with trapezoidal pitch profile, shown for in-phase stroking wing (ψ = 0°). The letters ‘f’ and ‘h’ denote the forewing and hindwing, respectively. The downstroke and upstroke intervals of forewing and hindwing for each flapping pattern are indicated at the bottom of the plot by solid and dashed lines, respectively.

The time history of the vertical force coefficient C_v_ of the corrugated wing and flat plate for the in-phase stroking wing with trapezoidal pitch profile is compared in Figure 15. Most of the vertical force is generated in the downstroke even in the case of a trapezoidal pitch profile. Comparing the forewing’s C_v_ of the corrugated wing and flat plate for in-phase stroking, it can be observed that the corrugated wing, indicated by black solid lines in Figure 15, generates more vertical force than the flat plate, indicated by red dotted lines in Figure 15, especially in the middle of the downstroke. In the case of the hindwing, the corrugated wing generates more vertical force than the flat plate in the downstroke interval (t/T = 0.1–0.3) and supination (t/T = 0.5–0.55), as indicated by black dash lines and red dash-dotted lines in Figure 15.

#### 3.2.3. Comparison of Flow Field for Corrugated Wing and Flat Plate with Trapezoidal Pitch Profile

**Figure 16:**
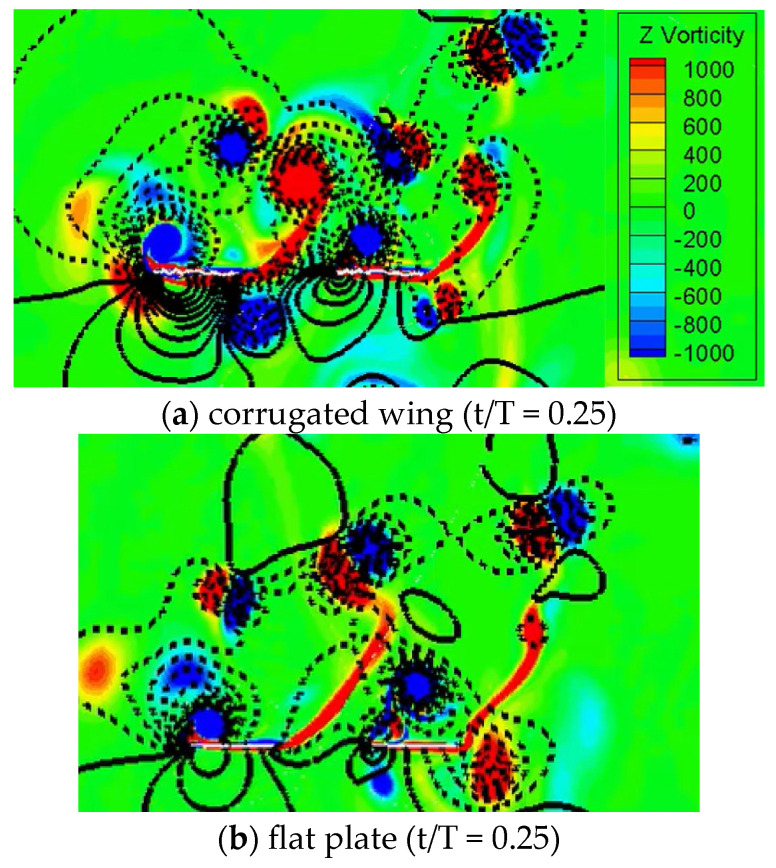
Comparison of flow field (vorticity and pressure isoline) for corrugated wing and flat plate with trapezoidal pitch profile at ψ = 0°. The positive pressure is shown by solid isoline while negative pressure is shown by dashed isoline.

The flow field of in-phase stroking for the corrugated wing and a flat plate at t/T = 0.25 are compared for the trapezoidal pitch profile in Figure 16. The pressure distribution is also illustrated as isolines in Figure 16. The positive pressure is given by solid isolines while the negative pressure is given by dash isolines in Figure 16. As indicated in Figure 15, the vertical force of the corrugated wing is larger than the flat plate, especially in the downstroke interval. Meanwhile, the detached CCWV on the lower surface for both wings are relatively stronger for the corrugated wing as compared to the flat plate, and hence, its vertical force is high for the corrugated wing. This detached CCWV exists as a result of wake capture and wing–wing interaction effects.

## 4. Conclusions

The effect of corrugated wings on the aerodynamic characteristics of a dragonfly-like hovering flapping wing is investigated using two-dimensional numerical simulations. Two types of pitch motion profiles, namely ‘sinusoidal’ and ‘trapezoidal’, are employed. The present simulations investigate the role of various flapping patterns (in-phase, 90° phase, and counter) for the corrugated wing and flat plate. The main findings of the study are as follows:

- The corrugated wing performs better than the flat plate in all three flapping patterns for both sinusoidal and trapezoidal pitch profiles. In the sinusoidal pitch profile, the vertical force generation of tandem wings is highest for in-phase stroking whereas it is lowest for counter stroking. In the trapezoidal pitch profile, the vertical force generation of tandem wings is highest for a 90° phase stroking whereas it is lowest for a counter stroking.
- In the sinusoidal pitch profile, the vertical force generation of tandem wing obtained for corrugated wing geometries is nearly 14%, 22%, and 12% higher than the flat plate geometries for ψ = 0°, 90°, and 180°, respectively.
- The corrugated wing sheds a relatively stronger detached CCWV vortex on the lower surface as compared to the flat plate, and hence, the vertical force is much higher for the corrugated wing.
- In the trapezoidal pitch profile, the vertical force generation of tandem wing obtained for corrugated wing geometries is nearly 27%, 22%, and 57% higher than the flat plate geometries for ψ = 0°, 90°, and 180°, respectively.
- By comparison, a flapping wing with sinusoidal pitch profile kinematics generates more vertical force than a flapping wing with trapezoidal pitch profile kinematics in all three flapping patterns.
- The delayed stall mechanism is further postponed in corrugated wing geometry as the corrugation shape traps the vortex structures which has a significant positive influence on the vertical force generation.

## Figures and Tables

**Table 1:** Various wing motion parameters.

Parameter	Value
thickness-to-chord ratio (t/c)	0.04
free-stream velocity U_∞_	0 m/s
Reynolds number Re	2150
flapping frequency f	40 Hz
mean angle of attack α_o_	45°
pitch amplitude B	45°
phase difference between the heave and pitch motion φ	0°
stroke plane inclination β	60°
stroke amplitude A_o_/c	2.5
phase difference between the forewing and the hindwing ψ	0°, 90°, and 180°
wing spacing (L/c)	2.1

**Table 2:** Results of mesh and time independence studies. (TW: tandem wing).

Case	Cells	Δt (s)	$\bar{C_{v}}$ (TW)
Medium M1	75,000	T/500	1.719
Fine M2	150,000	T/750	1.808
Refined M3	225,000	T/1000	1.847

## Data Availability

The data presented in this study are available on request from the corresponding author.

## Abbreviations

The following abbreviations are used in this manuscript:

MAV	Micro aerial vehicle
LE	Leading edge
PISO	Pressure Implicit with Split Operator algorithm
TW	Tandem wing
CWV	Clockwise vortex
CCWV	Counter clockwise vortex

## References

[1] Pan Y., Guo S., Whidborne J., Huang X. (2024). Aerodynamic performance of a flyable flapping wing rotor with dragonfly-like flexible wings. Aerosp. Sci. Technol..

[2] Yang X., Luo Y., Lang X., Wang W. (2024). Investigation of the aerodynamic performance of the dragonfly-inspired tandem wings considering the coupling between the stroke plane and phase difference. Aerosp. Sci. Technol..

[3] Sun W., Wang Y., He G., Wang Q., Yu F., Song W. (2024). Effects of kinematic parameters and corrugated structure on the aerodynamic performance of flexible dragonfly wings. J. Fluids Struct..

[4] Li H., Weigert S., Nabawy M.R. (2025). Controlling the utility of wake capture in hovering flapping flight: An experimental investigation. Aerosp. Sci. Technol..

[5] Addo-Akoto R., Yang H.H., Han J.S., Han J.H. (2023). Wing flexibility effect on aerodynamic performance of different flapping wing planforms. J. Fluids Struct..

[6] Lee H., Jang J., Lee S. (2020). An investigation of kinematic parameters and stroke function on stroke reversal for three-dimensional vortex structures around a flapping insect wing. Eur. J. Mech.-B/Fluids.

[7] Shanmugam A.R., Sohn C.H. (2019). Numerical investigation on thrust production and unsteady mechanisms of three-dimensional oscillating wing. J. Mech. Sci. Technol..

[8] Martínez-Muriel C., García-Villalba M., Flores O. (2024). On the role of wake-capture and resonance in spanwise-flexible flapping wings in tandem. J. Fluids Struct..

[9] Cong L., Teng B., Chen L., Bai W., Jin R., Chen B. (2023). Aerodynamic performance of low aspect-ratio flapping wing with active wing-chord adjustment. J. Fluids Struct..

[10] Lin T., Xia W., Pecora R., Wang K., Hu S. (2023). Performance improvement of flapping propulsions from spanwise bending on a low-aspect-ratio foil. Ocean Eng..

[11] Li G., Wu J., Zhang Y., Chen L. (2025). Unsteady Aerodynamic Performance of Tandem Configurations of Three Flapping and Fixed Airfoils. Aerosp. Sci. Technol..

[12] Li K., Zhou D., Sun X. (2023). Performance characteristics of flapping foil flow energy harvester that mimics movement of swimming fish. Ocean Eng..

[13] Bao T., Cao Y., Cao Y., Pan G., Lu Y., Xing C., Huang Q. (2024). Effects of motion parameters on the propulsion characteristics of flexible pectoral fins in bio-manta robots. Ocean Eng..

[14] Nguyen K., Park H.C. (2023). Feasibility study on mimicking the tail-beating supported gliding flight of Flying Fish. Ocean Eng..

[15] Chin D.D., Lentink D. (2016). Flapping wing aerodynamics: From insects to vertebrates. J. Exp. Biol..

[16] Shyy W., Kang C.K., Chirarattananon P., Ravi S., Liu H. (2016). Aerodynamics, sensing and control of insect-scale flapping-wing flight. Proc. R. Soc. A Math. Phys. Eng. Sci..

[17] Pesavento U., Wang Z.J. (2009). Flapping wing flight can save aerodynamic power compared to steady flight. Phys. Rev. Lett..

[18] Liu C., Shen T., Shen H., Lu B., Sun L., Chen G., Chi W. (2025). Mimicking Nature’s Insects: A Review of Bio-inspired Flapping-Wing Micro Robots (FWMRs). J. Bionic Eng..

[19] Liu F., Li S., Xiang J., Li D., Tu Z. A Dragonfly-inspired Flapping Wing Robot Mimicking Force Vector Control Approach. Proceedings of the 2024 IEEE International Conference on Robotics and Automation (ICRA).

[20] Jones S.K., Laurenza R., Hedrick T.L., Griffith B.E., Miller L.A. (2015). Lift vs. drag based mechanisms for vertical force production in the smallest flying insects. J. Theor. Biol..

[21] Weis-Fogh T. (1973). Quick estimates of flight fitness in hovering animals, including novel mechanisms for lift production. J. Exp. Biol..

[22] Lehmann F.O. (2004). The mechanisms of lift enhancement in insect flight. Naturwissenschaften.

[23] Ellington C.P. (1984). The aerodynamics of hovering insect flight. IV. Aerodynamic mechanisms. Philos. Trans. R. Soc. Lond. B Biol. Sci..

[24] Sane S.P. (2003). The aerodynamics of insect flight. J. Exp. Biol..

[25] Cheng X., Sun M. (2018). Very small insects use novel wing flapping and drag principle to generate the weight-supporting vertical force. J. Fluid Mech..

[26] Norberg R.Å. (1975). Hovering flight of the dragonfly Aeschna juncea L., kinematics and aerodynamics. Swimming and Flying in Nature.

[27] Wang Z.J. (2000). Two dimensional mechanism for insect hovering. Phys. Rev. Lett..

[28] Li D., Mu Y., Lau G.K., Chin Y., Lu Z. (2024). Numerical study on the aerodynamic performance of dragonfly (Anax parthenope julius) maneuvering flight during synchronized-stroking. Phys. Fluids..

[29] Li C., Dong H. (2017). Wing kinematics measurement and aerodynamics of a dragonfly in turning flight. Bioinspir. Biomim..

[30] Bode-Oke A.T., Zeyghami S., Dong H. (2018). Flying in reverse: Kinematics and aerodynamics of a dragonfly in backward free flight. J. R. Soc. Interface.

[31] Rüppell G. (1989). Kinematic analysis of symmetrical flight manoeuvres of Odonata. J. Exp. Biol..

[32] Wang Z.J. (2004). The role of drag in insect hovering. J. Exp. Biol..

[33] Hsieh C.T., Chang C.C., Chu C.C. (2009). Revisiting the aerodynamics of hovering flight using simple models. J. Fluid Mech..

[34] Hsieh C.T., Kung C.F., Chang C.C., Chu C.C. (2010). Unsteady aerodynamics of dragonfly using a simple wing–wing model from the perspective of a force decomposition. J. Fluid Mech..

[35] Sudhakar Y., Vengadesan S. (2010). Flight force production by flapping insect wings in inclined stroke plane kinematics. Comput. Fluids.

[36] Kim D., Choi H. (2007). Two-dimensional mechanism of hovering flight by single flapping wing. J. Mech. Sci..

[37] Bomphrey R.J., Nakata T., Henningsson P., Lin H.T. (2016). Flight of the dragonflies and damselflies. Philos. Trans. R. Soc. Lond. B Biol. Sci..

[38] Mitchell Z. (2018). Dragonfly locomotion: Ecology, form and function. Ph.D. thesis.

[39] Shumway N., Gabryszuk M., Laurence S. (2020). The impact of dragonfly wing deformations on aerodynamic performance during forward flight. Bioinspir. Biomim..

[40] Azuma A., Watanabe T. (1988). Flight performance of a dragonfly. J. Exp. Biol..

[41] Maybury W.J., Lehmann F.O. (2004). The fluid dynamics of flight control by kinematic phase lag variation between two robotic insect wings. J. Exp. Biol..

[42] Dickinson M.H., Lehmann F.O., Sane S.P. (1999). Wing rotation and the aerodynamic basis of insect flight. Science.

[43] Zheng Y., Wu Y., Tang H. (2016). An experimental study on the forewing–hindwing interactions in hovering and forward flights. Int. J. Heat Fluid Flow.

[44] Shanmugam A.R., Sohn C.H. (2019). Numerical investigation of the aerodynamic benefits of wing-wing interactions in a dragonfly-like flapping wing. J. Mech. Sci. Technol..

[45] Bie D., Li D. (2022). Numerical analysis of the wing–wake interaction of tandem flapping wings in forward flight. Aerosp. Sci. Technol..

[46] Nagai H., Fujita K., Murozono M. (2019). Experimental study on forewing–hindwing phasing in hovering and forward flapping flight. AIAA. J..

[47] Chen Z., Xie Y., Meng X. (2024). Unsteady aerodynamic forces of tandem flapping wings with different forewing kinematics. Biomimetics.

[48] He X., Wang C., Jia P., Zhong Z. (2024). The effect of hindwing trajectories on wake–wing interactions in the configuration of two flapping wings in tandem. Biomimetics.

[49] Wang J.K., Sun M. (2005). A computational study of the aerodynamics and forewing-hindwing interaction of a model dragonfly in forward flight. J. Exp. Biol..

[50] Gravish N., Peters J.M., Combes S.A., Wood R.J. (2015). Collective flow enhancement by tandem flapping wings. Phys. Rev. Lett..

[51] Meng X.G., Sun M. (2013). Aerodynamic effects of wing corrugation at gliding flight at low Reynolds numbers. Phys. Fluids.

[52] Hu H., Tamai M. (2008). Bioinspired corrugated airfoil at low Reynolds numbers. J. Aircr..

[53] Shi X., Huang X., Zheng Y., Zhao S. (2016). Effects of cambers on gliding and hovering performance of corrugated dragonfly airfoils. Int. J. Numer. Methods Heat Fluid Flow.

[54] Meng X.G., Xu L., Sun M. (2011). Aerodynamic effects of corrugation in flapping insect wings in hovering flight. J. Exp. Biol..

[55] Lian Y., Broering T., Hord K., Prater R. (2014). The characterization of tandem and corrugated wings. Prog. Aerosp. Sci..

[56] Flint T.J., Jermy M.C., New T.H., Ho W.H. (2017). Computational study of a pitching bio-inspired corrugated airfoil. Int. J. Heat Fluid Flow.

[57] Zhang Q., Xue R., Li H. (2023). Aerodynamic exploration for tandem wings with smooth or corrugated surfaces at low Reynolds number. Aerospace.

[58] Dao T.T., Loan Au T.K., Park S.H., Park H.C. (2020). Effect of wing corrugation on the aerodynamic efficiency of two-dimensional flapping wings. Appl. Sci..

[59] Chitsaz N., Siddiqui K., Marian R., Chahl J. (2022). Numerical and experimental analysis of three-dimensional microcorrugated wing in gliding flight. J. Fluids Eng..

[60] Chitsaz N., Siddiqui K., Marian R., Chahl J. (2021). An experimental study of the aerodynamics of micro corrugated wings at low Reynolds number. Exp. Therm. Fluid Sci..

[61] Hou D., Tan B., Shi B., Zhong Z. (2024). Aerodynamic effects of time-varying corrugations on dragonfly wings in flapping flight. Biomimetics.

[62] Broering T.M., Lian Y.S. (2012). The effect of phase angle and wing spacing on tandem flapping wings. Acta Mech. Sin..

[63] Sudhakar Y., Vengadesan S. (2010). The functional significance of delayed stall in insect flight. Numer. Heat Transf. Part A Appl..

[64] Bhat S.S., Zhao J., Sheridan J., Hourigan K., Thompson M.C. (2020). Effects of flapping-motion profiles on insect-wing aerodynamics. J. Fluid Mech..

[65] Kesel A.B. (2000). Aerodynamic characteristics of dragonfly wing sections compared with technical aerofoils. J. Exp. Biol..

[66] Lentink D.A., Dickinson M.H. (2009). Biofluid dynamic scaling of flapping, spinning and translating fins and wings. J. Exp. Biol..

[67] Han J.S., Chang J.W., Han J.H. (2016). The advance ratio effect on the lift augmentations of an insect-like flapping wing in forward flight. J. Fluid Mech..

[68] Han J.S., Chang J.W., Han J.H. (2017). An aerodynamic model for insect flapping wings in forward flight. Bioinspir. Biomim..

[69] Shanmugam A.R., Sohn C.H. (2019). Numerical investigation of the aerodynamic performance of dragonfly-like flapping foil in take-off flight. Proc. Inst. Mech. Eng. Part G. J. Aerosp. Eng..

[70] Srinidhi N.G., Vengadesan S. (2017). Ground effect on tandem flapping wings hovering. Comput. Fluids.

[71] Lua K.B., Lu H., Zhang X.H., Lim T.T., Yeo K.S. (2016). Aerodynamics of two-dimensional flapping wings in tandem configuration. Phys. Fluids.

[72] Wu D., Yeo K.S., Lim T.T. (2014). A numerical study on the free hovering flight of a model insect at low Reynolds number. Comput. Fluids.

